# Supplementary material for: Molecular basis for inhibiting human glucose transporters by exofacial inhibitors
Source: Nat Commun. 2022 May 12;13:2632. doi: 10.1038/s41467-022-30326-3 (PMC9098912; doi:10.1038/s41467-022-30326-3)
Supplement: Supplementary file 1 — Supplementary Information [file 41467_2022_30326_MOESM1_ESM.pdf]

Supplementary information for

Molecular basis for exofacial inhibitor of human glucose transporters

Nan Wang[1], Shuo Zhang[1], Yafei Yuan[1], Hanwen Xu[1], Elisabeth Defossa[2], Hans Matter[2], Melissa Besenius[2], Volker Derdau[2], Matthias Dreyer[2], Nis Halland[2], Kaihui Hu He[3], Stefan Petry[2], Michael Podeschwa[2], Norbert Tennagels[4], Xin Jiang[1,5,*] and Nieng Yan[1,6,*]

[1]State Key Laboratory of Membrane Biology, Beijing Advanced Innovation Center for Structural Biology, Tsinghua-Peking Joint Center for Life Sciences, School of Life Sciences, Tsinghua University, Beijing 100084, China. [2]Sanofi-Aventis Deutschland GmbH, R&D, Integrated Drug Discovery, Industriepark Höchst, 65926 Frankfurt am Main, Germany. [3]Sanofi-Aventis Deutschland GmbH, R&D, CMC Synthetics Early Development Analytics, Industriepark Höchst, 65926 Frankfurt am Main, Germany. [4]Bayer AG, R&D, Pharmaceuticals, TA Endocrinology, Metabolism & Reproductive Health, DIU Exploratory Pathobiology, 42096 Wuppertal, Germany. [5]School of Biotechnology and Biomolecular Sciences, the University of New South Wales, Sydney, NSW 2052, Australia. [6]Present address: Department of Molecular Biology, Princeton University, Princeton, NJ 08544, USA.

[*]To whom correspondence should be addressed: X. Jiang (xin.jiang@unsw.edu.au); N. Yan (nyan@princeton.edu).

This PDF file includes:

Supplementary Fig. 1-4

Supplementary Table 1-2

Supplementary Notes

Supplementary References

Supplementary Figures and Tables

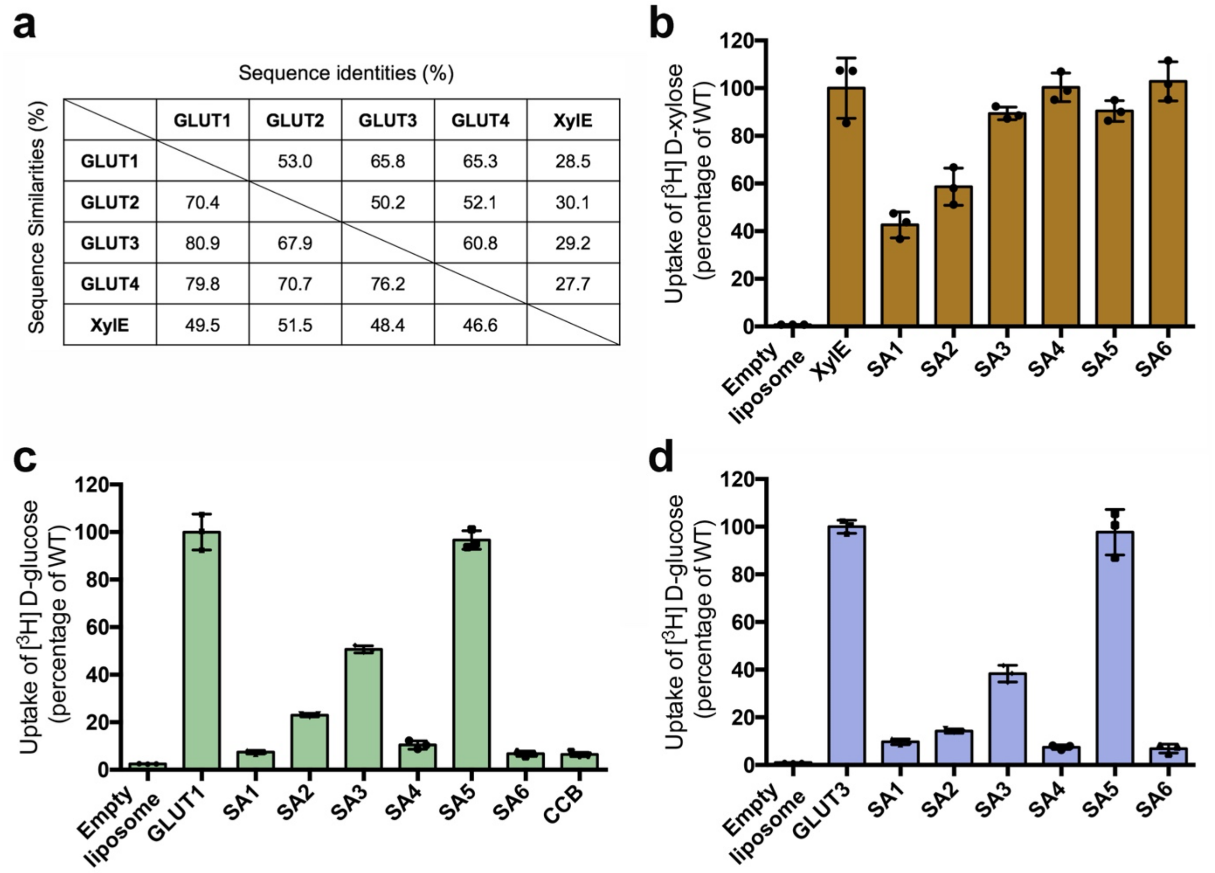

Sequence Similarities (%) \ Sequence identities (%)	GLUT1	GLUT2	GLUT3	GLUT4	XylE
GLUT1		53.0	65.8	65.3	28.5
GLUT2	70.4		50.2	52.1	30.1
GLUT3	80.9	67.9		60.8	29.2
GLUT4	79.8	70.7	76.2		27.7
XylE	49.5	51.5	48.4	46.6	

Supplementary Figure 1. Different inhibition patterns between XylE, GLUT1 and GLUT3.

a. Pairwise sequence alignment between GLUT1-GLUT4 and XylE. **b, c, d.** Inhibition of transport activities of XylE, GLUT1 and GLUT3 by SA compounds. The inhibition of isotope labeled substrate transport of XylE (**b**), GLUT1 (**c**) and GLUT3 (**d**) was examined in the presence of indicated inhibitors. Empty liposome refers to the protein-free liposome as control. The CCB was set as the positive control for proteoliposome-based inhibition assay. The final concentration of inhibitors for inhibition assay was 100 μM. The data represents mean ± SD for three independent experiments. Source data are provided as a Source Data file.

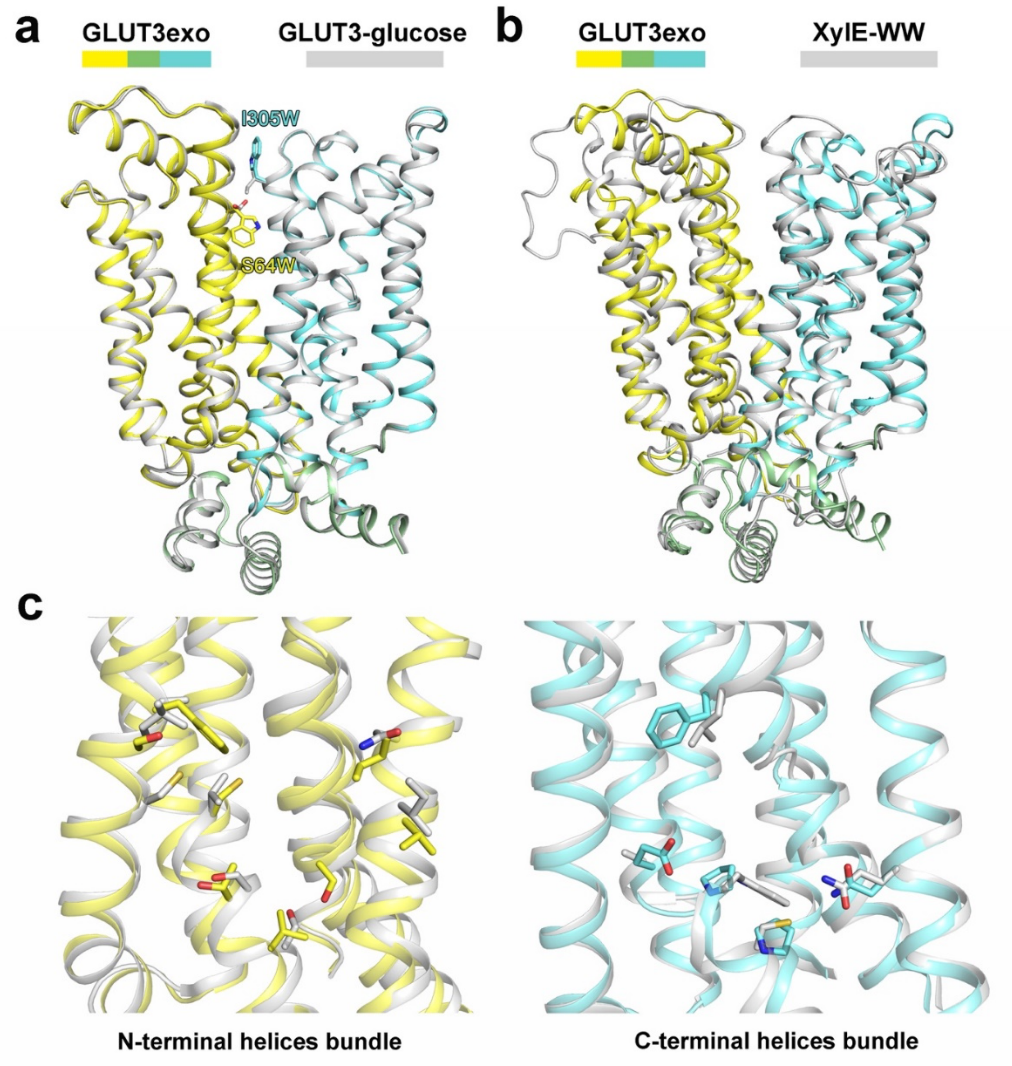

Supplementary Figure 2. Structural comparison between GLUT3exo, GLUT3-glucose and XylE-WW.

a. Structural alignment between GLUT3exo and GLUT3-glucose (PDB code: 4ZW9) complexes. The positions for double Trp mutations were shown as stick. **b.** Structural alignment between GLUT3exo and XylE-WW (PDB code: 6N3I) complexes. **c.** Structural alignment of residues in the central pocket between GLUT3exo and XylE-WW. The N- and C-terminal domains of GLUT3exo were colored yellow and cyan, respectively. XylE-WW was colored gray.

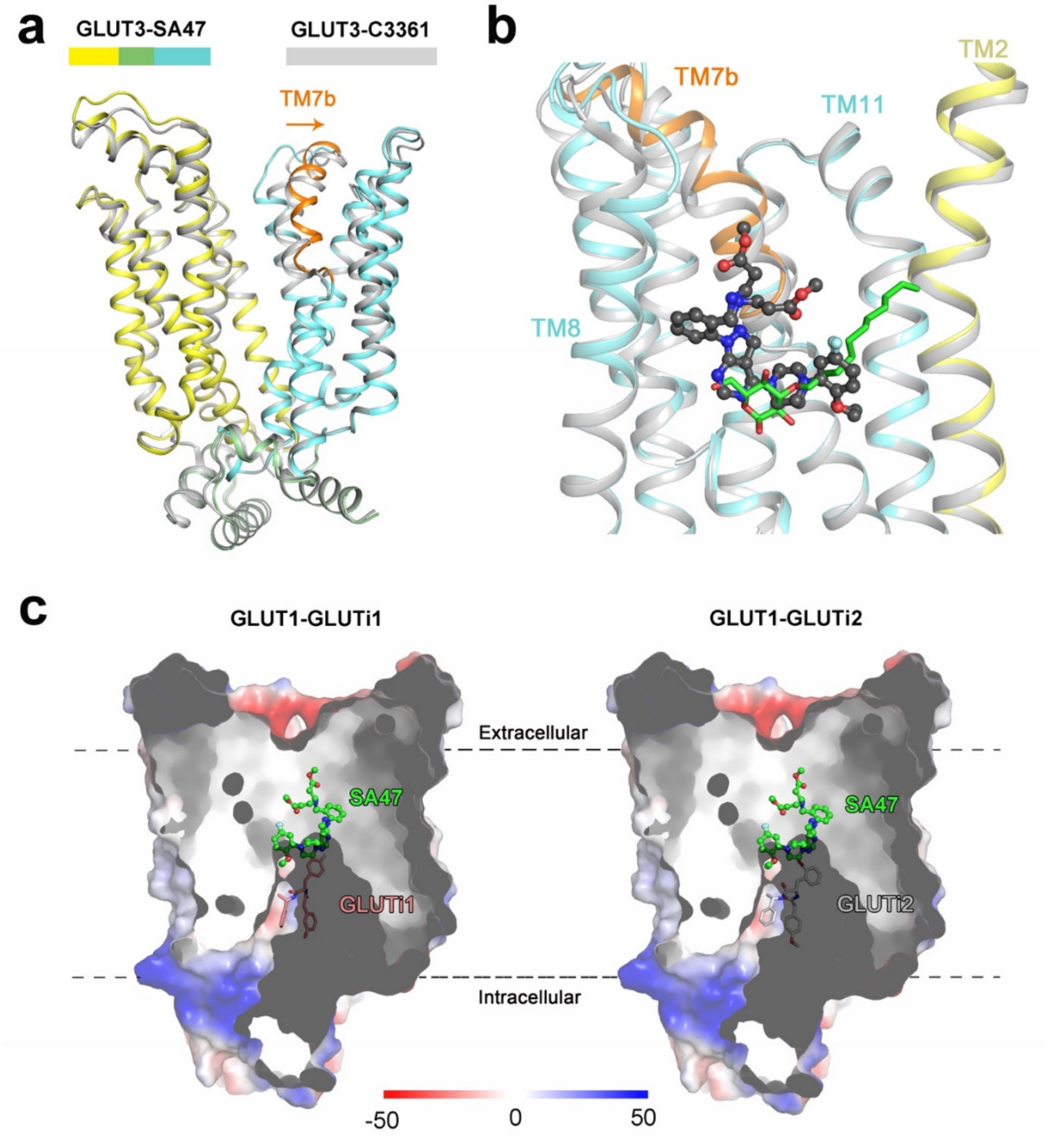

Supplementary Figure 3. Comparison of the endofacial and exofacial inhibitor binding pockets in GLUT1 and GLUT3 structures.

a. Structural alignment between GLUT3-SA47 and GLUT3-C3361 complexes. TM7b was highlighted in orange. The TM11 was omitted for a clear view of TM7b movement. **b**. Zoom in of the binding pockets of GLUT3-SA47 and GLUT3-C3361 (PDB code: 7CRZ) complexes. **c**. The inhibitor binding pockets of GLUT1-GLUTi1 (left) and GLUT1-GLUTi2 (right). The SA47 was positioned based on structural alignment between GLUT3-SA47 and GLUT1-GLUTi1/GLUTi2.

Supplementary Table 1. Summary of data collection and model statistics

	**GLUT3exo-glucose**	**GLUT3-SA47**
**Data collection**		
Number of crystals	**70**	**129**
Space group	**$P\ 2_1$**	**$P\ 2_1$**
Cell dimensions□□		
a, *b*, *c* (Å)	**48.23, 118.43, 53.73**	**77.59, 121.55, 95.87**
α, β, γ (°)	**90, 103.16, 90**	**90, 108.14, 90**
Resolution (Å)	**50 - 2.1 (2.2 - 2.1)**	**50 - 2.3 (2.4 - 2.3)**
R_{sym} or R_{merge}	**0.34 (1.47)**	**0.28 (2.45)**
$I / \sigma I$	**6.34 (1.45)**	**11.75 (1.07)**
$CC_{1/2}$ (%)	**98.0 (75.8)**	**99.8 (53.6)**
Completeness (%)	**99.9 (100)**	**99.9 (100)**
Redundancy	**13.49 (11.86)**	**23.07 (13.90)**
**Refinement**		
Resolution (Å)	**46.97 - 2.10**	**46.39 - 2.30**
No. reflections	**34,201**	**147,560**
R_{work} / R_{free}	**0.1767/0.2198**	**0.2119/0.2345**
**No. atoms**		
Protein	**3,621**	**7,212**
Ligand (Glucose/ SA47)	**12**	**152**
Others (Lipids, Waters)	**298**	**243**
***B*-factors**		
Protein	**30.08**	**54.53**
Ligand (Glucose, SA47)	**18.00**	**54.42**
Others (Lipids, Waters)	**40.84**	**60.31**
**R.m.s. deviations**		
Bond lengths (Å)	**0.007**	**0.007**
Bond angles (°)	**0.786**	**1.147**
**Ramachandran statistics**		
Favored regions (%)	**98.5**	**98.1**
Allowed regions (%)	**1.3**	**1.8**
Outliers (%)	**0.2**	**0.1**

R.m.s., root mean square. Values in parentheses are for the highest-resolution shell.

Supplementary Table 2. Microscale thermophoresis (MST) measurement table of the binding affinities between GLUT3 variants and SA47.

Binding moiety of SA47	GLUT3 mutants	Kd of SA47
	WT	316.5 ± 47.2 nM
aryl head [1]	F70A	1.5 ± 0.2 μM
	S71A	1.6 ± 0.4 μM
	N413A	809.7 ± 214.2 nM
piperazine moiety [2]	W386A	30.3 ± 10.7 μM
pyrazolopyrimidine core [3]	Q159A	4.1 ± 0.8 μM
	N315A	9.5 ± 2.2 μM
	E378A	1.1 ± 0.1 μM
pyrazoloaryl tail [4]	N32A	940.8 ± 213.3 nM
	F289A	163.8 ± 67.0 nM

Supplementary Notes

Chemical synthesis and characterizations

SA1, **SA2**, **SA3** and **SA4** are examples from Sanofi patent application WO19106122[1]:

	structure	example	m/z	m/z calc.
SA1	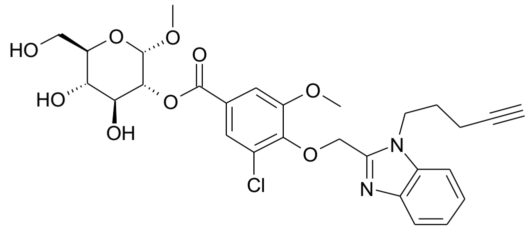	186	575.2/577.2 [M+H]$^+$	575.18
SA2	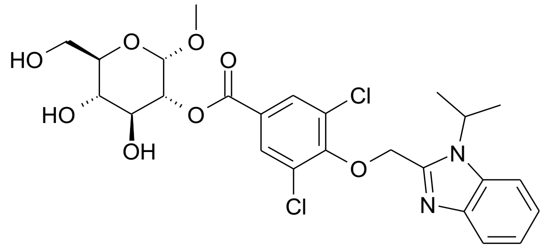	115	555.12 [M+H]$^+$	555.13
SA3	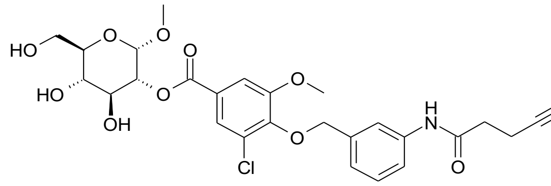	78	564.2/565.9 [M+H]$^+$	564.16
SA4	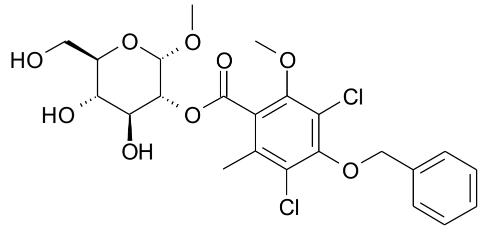	153b	561.1 [M-H+formic acid]$^-$	561.09

SA5

SA 5 is a screening hit from Sanofi library.

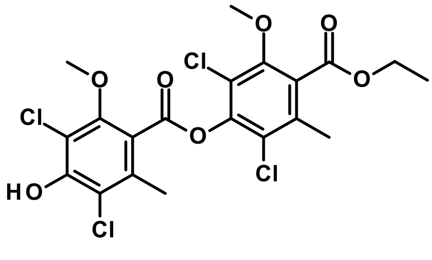

LC/MS: m/z = 509.17 [M-H]$^-$; m/z calc = 508.97

Syntheses of compounds SA6 and SA47

1: X1=Br, X2=H, R=F; **2**: X1=H, X2=CN, R=H

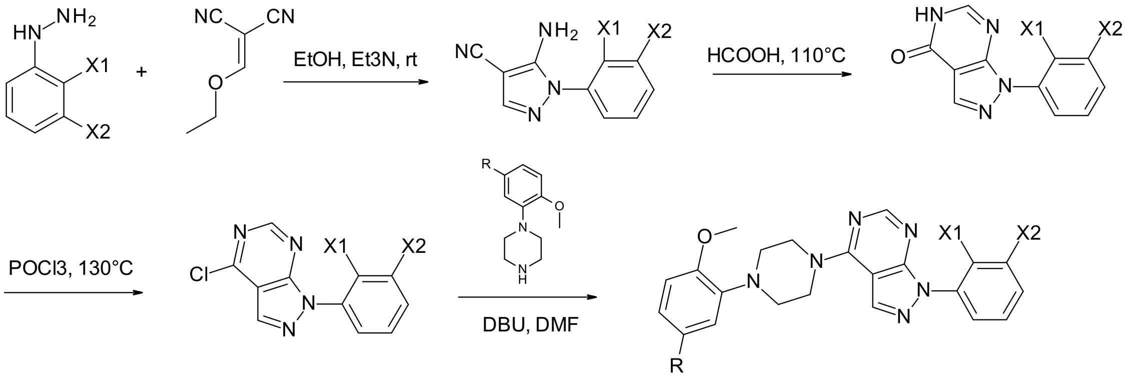

1 and 2

Compounds **1** and **2** were synthesized according the procedures described in PCT Int. Appl. 2013182612[2] and previously reported method[3].

SA6

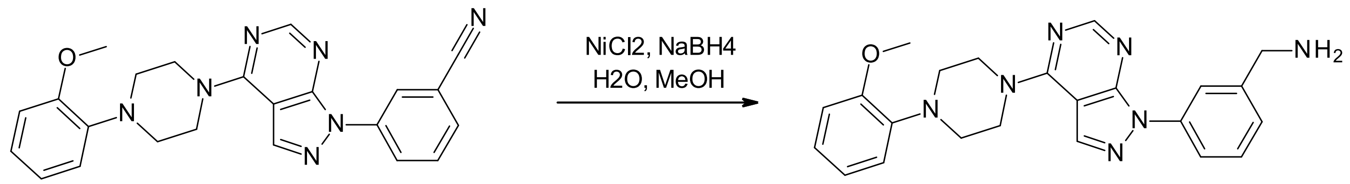

2 **SA6**

To a suspension of 145 mg (0.35 mmol, 1 eq.) cyanide **2** (3-(4-(4-(2-methoxyphenyl)-piperazin-1-yl)-1H-pyrazolo[3,4-d]pyrimidin-1-yl)benzonitrile), 8.5 mg (0.035 mmol) nickel(II) chloride hexahydrate and 146 µL (1.06 mmol) water in methanole (25 mL) 160 mg (4.2 mmol) sodium borohydride were added over a period of seven days. The reaction mixture was stirred at room temperature. After filtration the solid residue was dissolved in DMSO, filtered and purified by HPLC to give 8 mg (3.5% yield) of the desired product.

LC/MS: m/z = 416.2 $[M+H]^+$; m/z calc = 416.22

^{1}H NMR (400.23 MHz, DMSO-d_6) d ppm 8.70 (s, 1 H), 8.44 (s, 1 H), 8.28 (m, 4 H), 7.63 (t, J=7.89, 7.89 Hz, 1 H), 7.45 (d, J=7.70 Hz, 1 H), 6.97 (m, 4 H), 4.15 (m, 6 H), 3.84 (s, 3 H), 3.16 (m, 4 H)

SA47

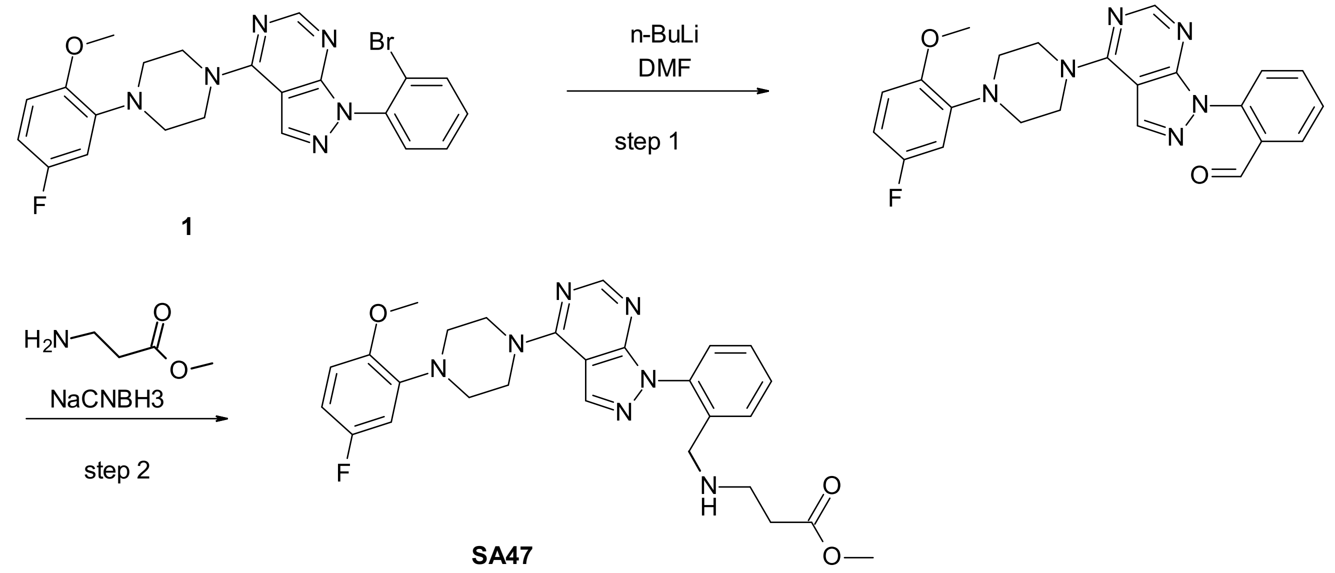

1

SA47

Step 1, aldehyde:

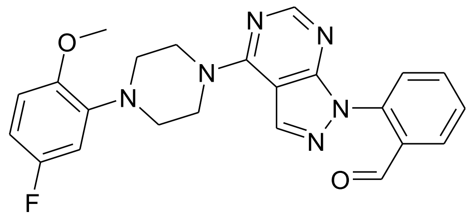

3.00 g (6.21 mmol, 1.0 eq) of bromide **1** was dissolved in 40 mL tetrahydrofuran and under argon atmosphere cooled to -78°C in a ethanol/dry ice cooling bath. Then 6 mL n-BuLi (9.31 mmol, 1.6 M in heptane, 1.5 eq) was added dropwise via syringe. The solution was stirred for 30 min and then 906 mg (12.4 mmol, 2.0 eq) dimethylformamide was added dropwise. The reaction mixture was stirred for two hours and then the cooling bath was removed. After the reaction mixture has come to rt 2 mL of 1N HCl was added and then the solvent was removed in vacuo. The residue was purified by reversed phase HPLC to give 1.47 g (3.42 mmol, 55%) of the aldehyde.

Step 2, reductive amination

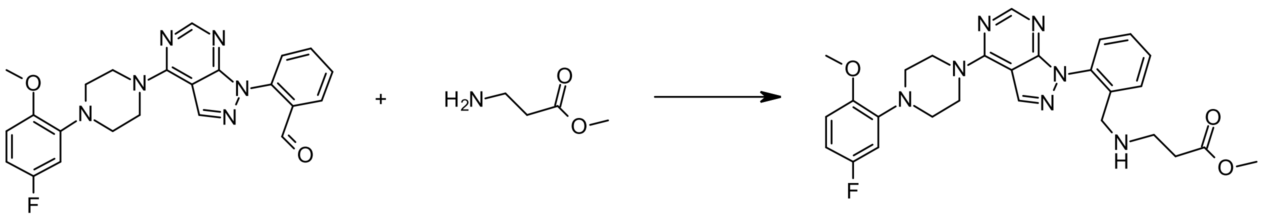

86.0 mg (0.20 mmol, 1.0 eq) of aldehyde and 28.0 mg (0.20 mmol, 1.0 eq) beta-Alaninmethylester x HCl was dissolved in 10 mL tetrahydrofuran and stirred at room temperature with 20 mg molecular sieves (4 Å) for 2 h. The solution was filtered and then 16.0 mg (0.20 mmol, 1.3 eq) sodiumcyanoborohydride were added as solid. The reaction was stirred at room temperature for 4 h and then 1 mL water was added. The solvent was evaporated to dryness, the residue dissolved in DMF and purified by prep. RP HPLC. After lyophilization the corresponding TFA product was isolated as colorless solid to give 57.0 mg (0.09 mmol, 46%) of the product.

LC/MS: m/z = 520.33 $[M+H]^+$; m/z calc = 520.25

^{1}H NMR (400.23 MHz, DMSO-d_6) d ppm 8.94 (br s, 2 H), 8.73 (s, 1 H), 8.39 (s, 1 H), 7.78 (d, J=6.56 Hz, 1 H), 7.65 (s, 2 H), 7.64 (m, 1 H), 6.99 (dd, J=9.54, 5.38 Hz, 1 H), 6.80 (m, 2 H), 4.17 (m, 4 H), 4.11 (br s, 2 H), 3.83 (s, 3 H), 3.64 (s, 3 H), 3.56 (m, 3 H), 3.19 (m, 6 H), 2.76 (t, J=6.97, 6.97 Hz, 2 H)

Supplementary References

1 Petry, S. *et al.* Preparation of aminoglycoside and peptide conjugates of a pharmaceutical agent and a moiety capable of binding to a glucose sensing protein. WO2019106122 (2019).

2 Heisler, I. *et al.* Glucose transport inhibitors. PCT/EP2013/061618 (2013).

3 Siebeneicher, H. *et al.* Identification of novel GLUT inhibitors. *Bioorg Med Chem Lett* **26**, 1732-1737, doi:10.1016/j.bmcl.2016.02.050 (2016).
